# Supplementary figures and images for: Association between Smoking Status and Incident Non-Cystic Fibrosis Bronchiectasis in Young Adults: A Nationwide Population-Based Study
Source: J Pers Med. 2022 Apr 26;12(5):691. doi: 10.3390/jpm12050691 (PMC9144886; doi:10.3390/jpm12050691)

Figure S1: Study periods and follow up

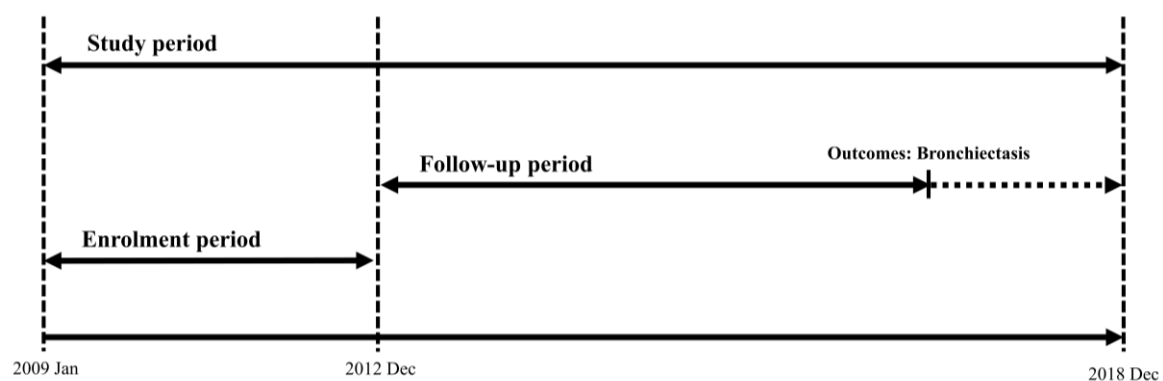

Supplement: Supplementary file 1 [file jpm-12-00691-s001.zip › jpm-1674452-supplementary.pdf]
